# Supplementary material for: Splice-Junction-Based Mapping of Alternative Isoforms in the Human Proteome
Source: Cell Rep. Author manuscript; Available in PMC 2020 Jan 15. (PMC6961840; doi:10.1016/j.celrep.2019.11.026)
Supplement: 3 [file NIHMS1546469-supplement-3.zip › DF2/PXD0010154/Lung-218-Q96RU3-NKGATPEDFSNLPPEQR.pdf]

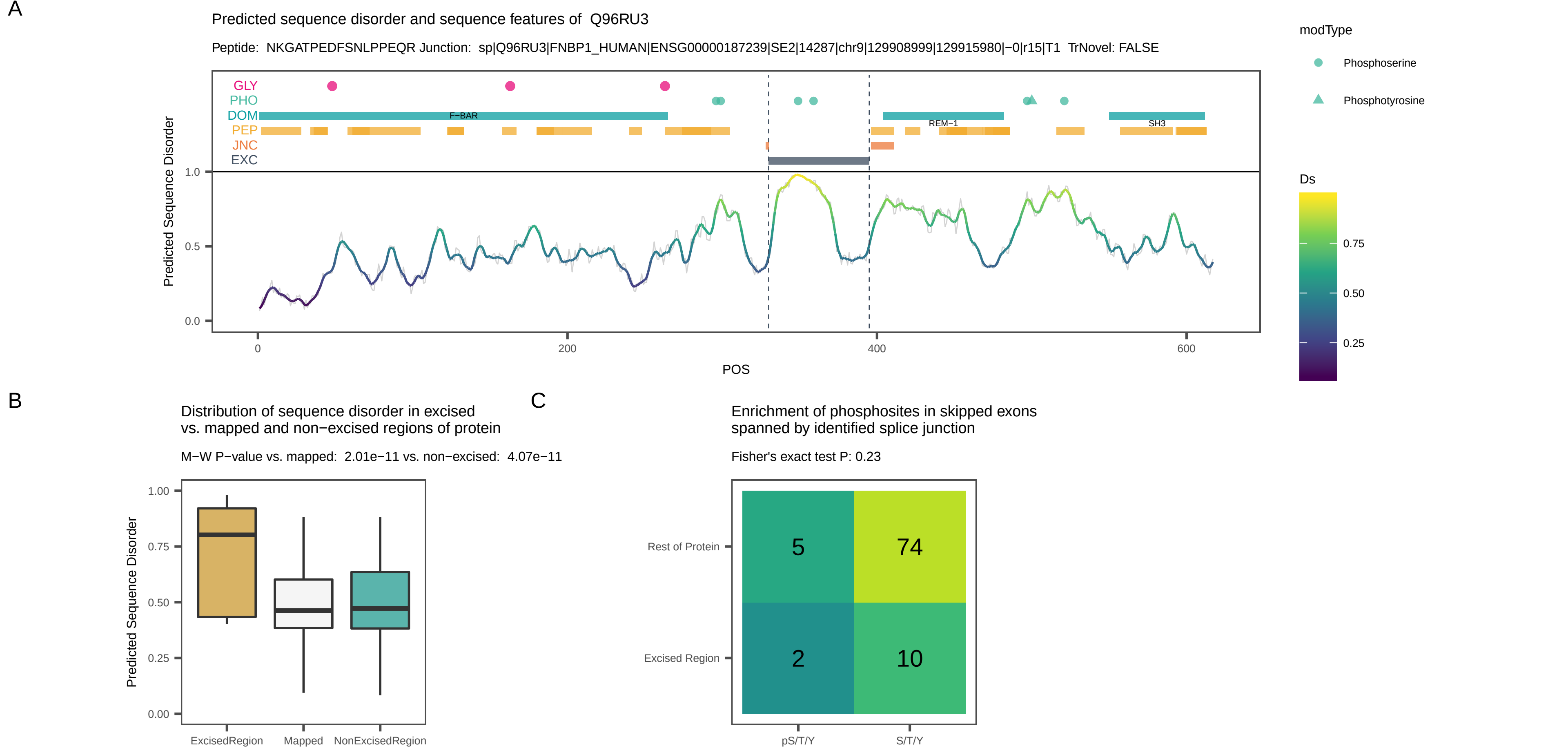
A
Predicted sequence disorder and sequence features of Q96RU3
Peptide: NKGATPEDFSNLPPEQR Junction: sp|Q96RU3|FNBP1_HUMAN|ENSG00000187239|SE2|14287|chr9|129908999|129915980|−0|r15|T1 TrNovel: FALSE
GLY
PHO
DOM
PEP
JNC
EXC
F−BAR
REM−1
SH3
Predicted Sequence Disorder
1.0
0.5
0.0
0
200
400
600
POS
modType
Phosphoserine
Phosphotyrosine
Ds
0.75
0.50
0.25
B
Distribution of sequence disorder in excised
vs. mapped and non−excised regions of protein
M−W P−value vs. mapped: 2.01e−11 vs. non−excised: 4.07e−11
Predicted Sequence Disorder
1.00
0.75
0.50
0.25
0.00
ExcisedRegion
Mapped
NonExcisedRegion
C
Enrichment of phosphosites in skipped exons
spanned by identified splice junction
Fisher's exact test P: 0.23
Rest of Protein
Excised Region
5
74
2
10
pS/T/Y
S/T/Y
